# Robot-assistant visualized minimally invasive aspiration (RAVMIA) technique for intracerebral hemorrhage evacuation: Case series

**DOI:** 10.1016/j.heliyon.2024.e39803

**Published:** 2024-10-24

**Authors:** Zhenyu Luo, Chen Li, Xiaoguang Du, Tingzhong Wang

**Affiliations:** Department of Neurosurgery, Shandong Provincial Third Hospital, Cheeloo College of Medicine, Shandong University, Jinan, Shandong, China

**Keywords:** Intracerebral hemorrhage, Robot surgery, Endoscopy, Minimally invasive, Case report

## Abstract

**Background and Importance:**

The surgical management of intracerebral hemorrhage (ICH) remains controversial due to unfavorable outcomes reported in several influential clinical trials. There is a pressing need for novel instrumentation and approaches that optimize evacuation efficiency while minimizing invasiveness. Among the emerging techniques, endoscopic surgery and robot-assisted minimally invasive catheterization (robotic MISTIE) show potential. However, the former still results in brain damage at a centimeter level, while the latter exhibits low evacuation efficiency due to its non-visualized nature.

**Methods:**

We have developed a novel technique called robot-assisted visualized minimally invasive aspiration (RAVMIA) for the evacuation of ICH. This technique integrates neurosurgical robot navigation, contact-visible endoscopy, and minimally invasive catheterization. The efficacy of RAVMIA was evaluated using robotic MISTIE as a historical control.

**Results:**

The RAVMIA technique was successfully implemented in three cases of ICH without complications. Brain damage was limited to 5mm. Compared to robotic MISTIE, RAVMIA did not prolong operative time (20.67 ± 4.04 minutes vs. 20.87 ± 5.74 minutes, p = 0.946) but significantly increased the intraoperative hematoma evacuation rate from 80.8 ± 4.1 % to 86.6 ± 1.3 % (p = 0.003). Consequently, the end-of-treatment residual ICH volume decreased from 5.3 ± 2.95 ml to 1.3 ± 1.05 ml (p = 0.004), and the hospital stay was reduced from 12.87 ± 4.55 days to 10.67 ± 4.04 days (p = 0.029).

**Conclusion:**

The preliminary application of the RAVMIA technique demonstrates its safety and feasibility in treating long, oval-shaped basal ganglia hematoma and brain stem hematoma. This method achieves high evacuation efficiency while minimizing invasiveness. Further technical optimization and clinical trials are warranted to fully explore its potential.

## Abbreviations:

CT: computed tomography
EOT: end of treatment
ES: endoscopic surgery
GCS: Glasgow coma scale score
ICH: intracerebral hemorrhage
ICP: intracranial pressure
MISTIE: minimally in\vasive surgery with thrombolysis in intracerebral hemorrhage evacuation
NIHSS: the national institutes of health stroke scale score
RAVMIA: robot-assisted visualized minimally invasive aspiration
RCT: randomized controlled trial
SCUBA: stereotactic intracerebral hemorrhage underwater blood aspiration
STICH: international surgical trial in intracerebral hemorrhage

## 1 Introduction

The incidence of spontaneous intracerebral hemorrhage (ICH) has been rising over the past 20 years [1]. The role of surgery for ICH has been controversial for many years. The deep location of the ICH necessitates brain tissue damage during its evacuation. Neither conventional craniotomy nor minimally invasive catheterization has proven superior to conservative treatment in clinical trials [[2], [3], [4]]. Technically, the former shows better hematoma evacuation but causes significant brain damage. The latter minimizes brain damage but has lower evacuation efficacy due to lack of hematoma visibility. Thus, achieving maximum hematoma evacuation with minimal brain damage is a key goal for ICH surgery.

Sheath-mediated endoscopic surgery (ES) allows effective ICH evacuation under direct visualization with reduced invasiveness to brain parenchyma. Preliminary clinical studies have shown its advantages [5]. However, this technique still causes about 1–3 cm of brain damage. Multi-center clinical trials are being conducted to confirm the superiority of sheath-mediated ES for ICH evacuation [6,7]. Meanwhile, new instruments and approaches are being developed to achieve visualized hematoma evacuation with minimal invasiveness. For example, the SCUBA technique uses wet-field rigid endoscopy to remove the ICH under direct visualization with millimeter-level brain damage [8]. In this paper, brain damage refers to the extent of artificial disruption or disturbance to normal brain tissue required to access the deep hematoma. In various minimally invasive procedures, this specifically refers to the outer diameter of the drainage tube, trocar, or sheath.

In recent years, neurosurgical robots have been used to facilitate minimally invasive catheterization. The precise target and trajectory control provided by the robots significantly enhance drainage efficiency [9]. However, its non-visualized nature remains unchanged. In this article, we present a novel technique called robot-assisted visualized minimally invasive aspiration (RAVMIA) for ICH evacuation. This technique combines neurosurgical robot navigation, contact-visible endoscopy, and minimally invasive catheterization to achieve visualized hematoma evacuation with millimeter-level brain damage. We also demonstrate its successful application in treating ICHs located in the basal ganglia and brain stem.

## 2 Methods

### 2.1 Patients

A series of consecutive ICH patients between December 2023 and March 2024 at our institute were retrospectively analyzed. The inclusion criteria were as follows: (1) 30–60 ml long oval-shaped basal ganglia hematoma without any signs of brain herniation, or (2) brain stem hemorrhage greater than 5 ml with a GCS less than 8. The exclusion criteria were as follows: (1) secondary ICH caused by aneurysm, vascular malformation, Moyamoya disease, or cavernoma; (2) basal ganglia hematoma with a scattered shape. All patients underwent a one-stop CT (CT, CTA, CTP) in the emergency room to rule out the aforementioned structural vascular abnormalities. In total, 18 patients were enrolled in this study. The first 15 patients underwent robotic MISTIE, while the last 3 underwent RAVMIA. All patients underwent an immediate postoperative CT scan to evaluate the residual hematoma, followed by admission to the NICU for life support, with systolic blood pressure maintained under 140 mmHg. Fibrinolytic therapy was started at the earliest 6 hours after ICH onset. 40,000 IU of urokinase (Tianjin Biochem Pharmaceutical Co. Ltd., Tianjin, China) dissolved in 2 ml saline was injected twice daily into the residual hematoma cavity through the drainage tube, which was then occluded for 2 hours and reopened to release the lytic hematoma. The frequency of follow-up CT scans was determined based on the volume of residual hematoma observed in the initial postoperative CT and the daily drainage amount. The drainage tube was removed under the following conditions: (1) the residual hematoma within the reach of the drainage tube was less than 3 ml; (2) CT showed that even after retracting the drainage tube, it could not remain within the residual hematoma; (3) although the drainage tube was within the residual hematoma cavity, no drainage fluid flowed out within 2 days after fibrinolytic therapy; (4) the drainage tube had been in place for 7 days.

### 2.2 The RAVMIA technique

#### 2.2.1 Robot navigation

After administering general anesthesia, the patient's head is securely connected to a neurosurgical robot (Remebot® RM-100, Remebot Technology Co., Beijing, China) using a three-pin head frame. Thin-slice CT images in DICOM format are imported into the robot's software system for image processing and surgical planning. The system performs a three-dimensional reconstruction of the hematoma and calculates its volume. The deepest point of the hematoma is designated as the target site. An entry site on the scalp is adjusted to align the surgical trajectory with the long axis of the oval-shaped hematoma. The surgical trajectory approximately follows the midline of the hematoma in axial, sagittal, and coronal slices. A surgical plan is established. The hematoma is divided into two layers—top and bottom—using the midpoint of the long axis as the boundary. The patient's forehead and nasal bridge are scanned using a laser pen. Registration is accomplished through a laser point cloud methodology. After completing registration, the robotic arm automatically moves above the entry site along the preplanned trajectory. All subsequent surgical instruments, including the drill, trocar, endoscope, biopsy needle, and drainage tube, are inserted into the working channel of the robotic arm. The robot automatically regulates the depth of these instruments.

#### 2.2.2 Minimally invasive catheterization and contact-visible endoscopy

**Fig. 1:**
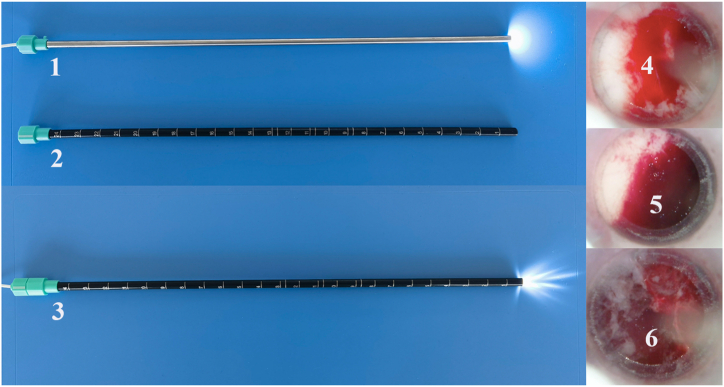
The contact-visible endoscope and hematoma characteristic categories. A conventional endoscope (1) is encased in a light-blocking sheath with a transparent distal end (2) to create a contact-visible endoscope (3). Based on visual observations through endoscopy, hematomas are classified into three categories: liquid-like (4), colloid-like (5), and solid-like (6).

The scalp is incised by 1 cm at the robot-defined entry site. A 5 mm electric drill is used to penetrate the skull. A 5 mm metal trocar, guided by a Kirschner wire, is inserted into the bone hole to pierce the dura mater. The trocar is positioned at the roof of the bottom layer, and the Kirschner wire is then removed. All subsequent instruments are introduced and withdrawn through the working channel of the robotic arm and the trocar. A disposable contact-visible endoscope (QKⅢC-13070, Kezhong Medical Technology Co., Changsha, China) is inserted to the target site in the bottom layer and connected to a laptop computer for direct visualization. Unlike conventional distance-visible endoscopy, which offers visibility over a range of distances, contact-visible endoscopy is designed to visualize tissue structures even when the lens is in direct contact with the tissue. This is achieved by incorporating a sleeve with a transparent distal end onto the conventional endoscope. Based on the appearance observed by the endoscopy, hematomas are categorized as liquid-like, colloid-like, and solid-like (Fig. 1). Liquid-like and colloid-like hematomas should be thoroughly aspirated during surgery, while a small amount of liquid-like hematoma can be drained postoperatively. The solid-like hematoma, which mainly consists of residual hematoma, requires intraoperative targeted aspiration and postoperative drainage. In the bottom layer of the hematoma, aspiration is performed using a multi-hole drainage tube after determining the hematoma's characteristics through endoscopy. Endoscopy is then used to assess the efficacy of hematoma clearance. If satisfactory, the trocar is repositioned at the roof of the top layer, and the aforementioned procedure is repeated in the top layer of the hematoma. The endoscope is then positioned at the target site and gradually withdrawn along its entire trajectory for a comprehensive examination of solid-like residual hematoma. The depth and orientation of the residual hematoma are meticulously documented, followed by targeted aspiration using a biopsy needle with a unilateral hole. Finally, upon removal of the trocar, a drainage tube is carefully retained at the precise depth of the residual hematoma. Video 1 illustrates the procedural concept of the RAVMIA technique.

#### 2.2.3 The illustrative clinical cases

##### 2.2.3.1 Case 1

**Fig. 2:**
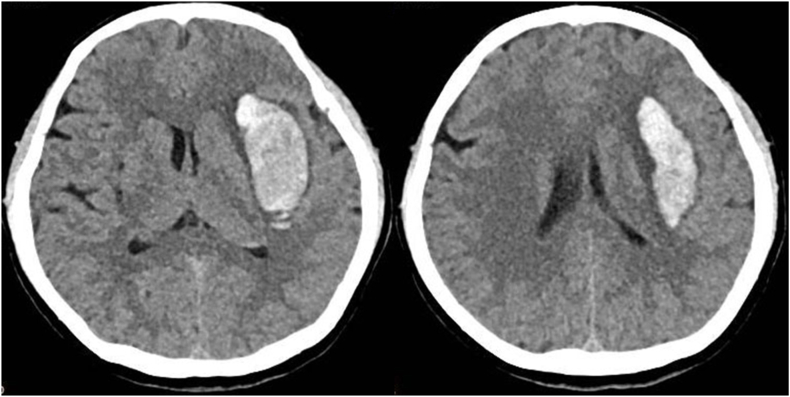
The preoperative CT of case 1.

**Fig. 3:**
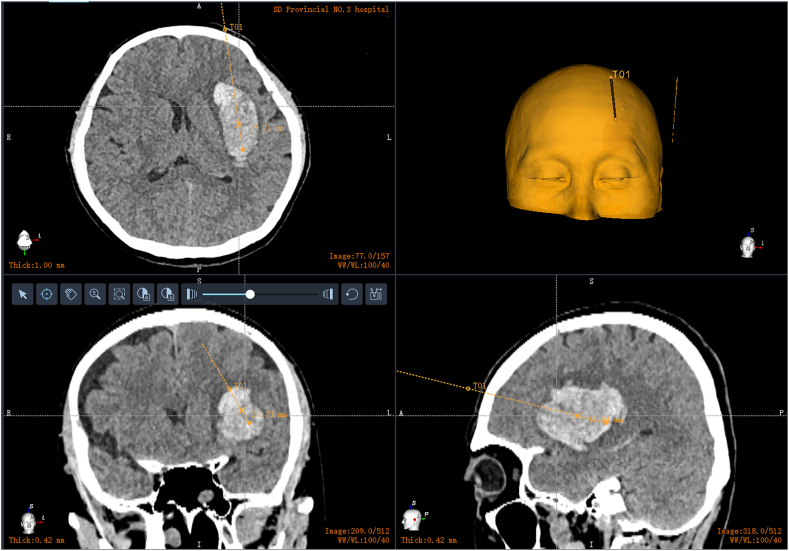
The surgical plan generated by the robot. A surgical trajectory was planned along the longitudinal axis of the hematoma. This trajectory closely approximated the midline in the axial, sagittal, and coronal planes.

**Fig. 4:**
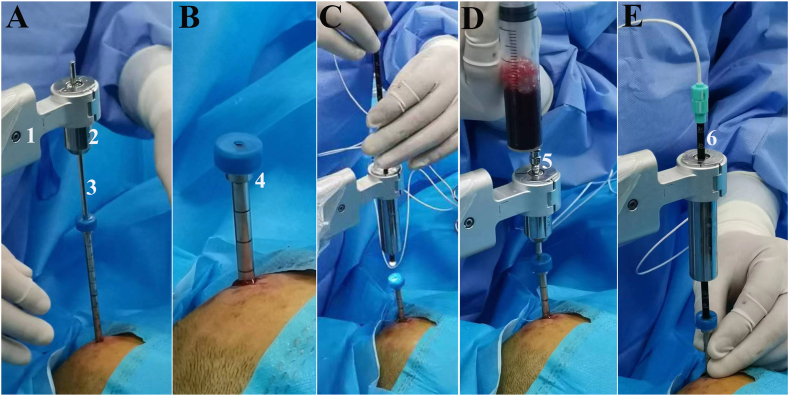
The actual procedure of RAVMIA. The robot-defined entry site was used to create a 1 cm scalp incision and a 5 mm bur hole. A 5 mm metal trocar, guided by a Kirschner wire, was inserted to access the hematoma (A and B). After confirming the hematoma's characteristics through contact-visible endoscopy (C), aspiration was performed (D). Endoscopy was then used to precisely determine the depth and orientation of the residual hematoma, guiding subsequent aspiration procedures (E). (1 robotic arm, 2 working channel, 3 Kirschner wire, 4 trocar, 5 biopsy needle, 6 contact-visible endoscope).

**Fig. 5:**
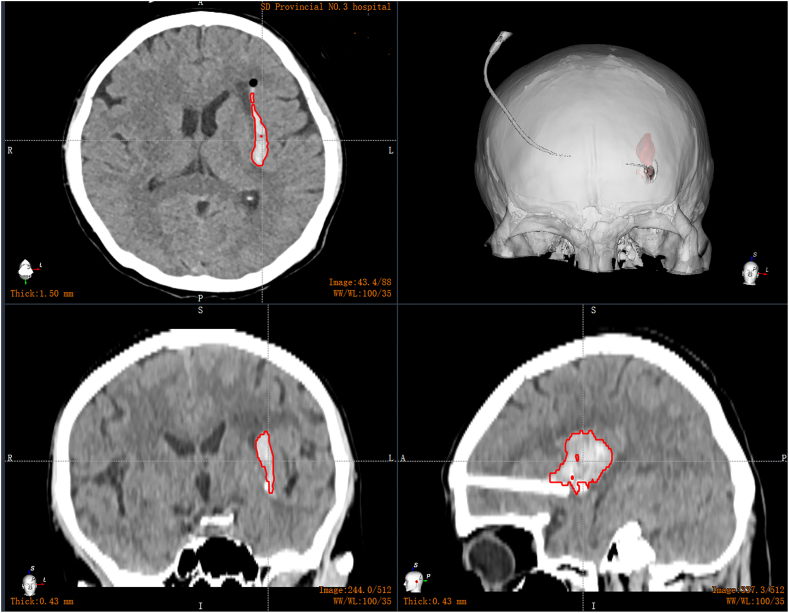
The postoperative CT of case 1.

A 54-year-old female presented with sudden right hemiplegia and dysphonia lasting for 2 hours. She had a history of hypertension and irregularly took oral antihypertensive medications. Her blood pressure was 190/95 mmHg, heart rate 120 bpm, and SpO_2_ 95 %. Although conscious, she was unable to articulate complete sentences. Both pupils were equal with a diameter of 3 mm. Muscle strength in her right limbs was assessed as grade 0. A CT scan revealed an elongated hyperdense lesion in the left basal ganglia (Fig. 2). This led to a diagnosis of ICH. Considering its lateral location in the putamen, rapid evacuation of the ICH could potentially alleviate her symptoms. After obtaining her consent, we performed RAVMIA. The patient was positioned supine. A surgical trajectory was planned along the longitudinal axis of the hematoma (Fig. 3). The procedure began with an incision on the left forehead (Fig. 4). The contact-visible endoscopy revealed the colloid-like characteristic of the bottom layer hematoma (Video 2). After the hematoma was aspirated, endoscopic examination confirmed successful evacuation (Video 3). The procedure was repeated for the top layer hematoma. The entire trajectory endoscopy revealed solid-like residual hematoma at the roof of the top layer and at a depth of 51 mm (Video 4). A biopsy needle was inserted into these specific depths for targeted aspiration. A drainage tube was placed at 51 mm. A postoperative CT scan demonstrated successful evacuation of the hematoma (Fig. 5). The drainage tube was removed on postoperative day two. The patient was alert and oriented. Muscle strength in the right lower limb had improved to grade III. Her expressive language function showed significant progress, enabling her to articulate simple sentences.

##### 2.2.3.2 Case 2

**Fig. 6:**
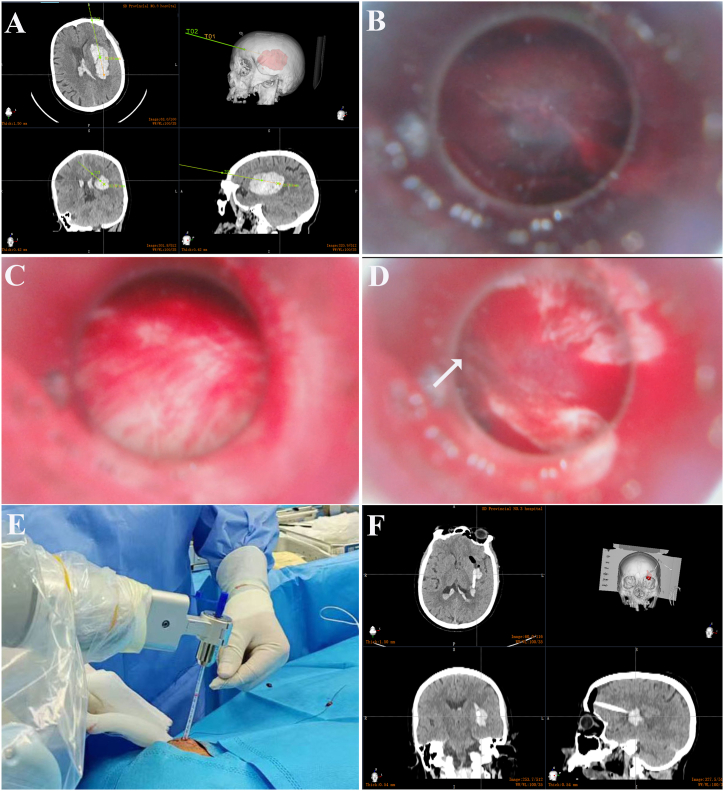
The RAVMIA technique for case 2. The surgical trajectory along the longitudinal axis of the hematoma was established by the robot (A). Endoscopy revealed a solid-like hematoma in the bottom layer (B), which was completely evacuated through aspiration (C). The same procedure was repeated in the top layer. The entire trajectory endoscopy revealed a residual hematoma at a depth of 77 mm and oriented at 9 o'clock (D). Targeted aspiration was performed using a biopsy needle, followed by the placement of a drainage tube (E). A postoperative CT scan confirmed the successful evacuation of the hematoma (F). (arrow: residual hematoma).

A 72-year-old female presented with right extremity weakness lasting for 2 hours. She gradually developed symptoms including nausea, vomiting, slurred speech, and confusion. She had a history of hypertension, diabetes, stroke, and coronary artery stenting. She was taking aspirin. Upon arrival at the emergency room, her blood pressure was 203/85 mmHg. Her Glasgow Coma Scale (GCS) score was 10. Both pupils were sensitive to light reflexes. Muscle strength in the right limb was graded as zero. A CT scan revealed an elongated, oval-shaped ICH in the left basal ganglia (Fig. 6A). After obtaining consent, a RAVMIA procedure was performed (Fig. 6B and C). The endoscopy revealed a residual hematoma at a depth of 77 mm (Fig. 6D), where a drainage tube was retained (Fig. 6E). A postoperative CT scan confirmed successful evacuation of the hematoma (Fig. 6F). The drainage tube was removed on postoperative day two. The patient remained alert and capable of speaking simple sentences. Muscle strength in the right lower limb improved to grade III.

##### 2.2.3.3 Case 3

**Fig. 7:**
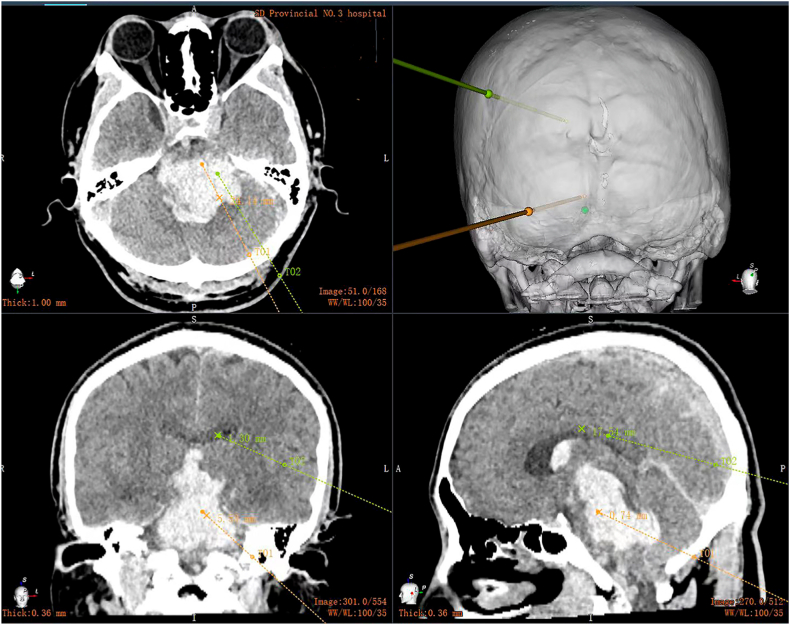
The surgical plan of case 3. The robotic system established a trans-medipeduncle intraparenchymal surgical trajectory from the suboccipital paramedian area to the hematoma. Additionally, an external ventricular drainage of the occipital horn was designed for ICP monitoring.

**Fig. 8:**
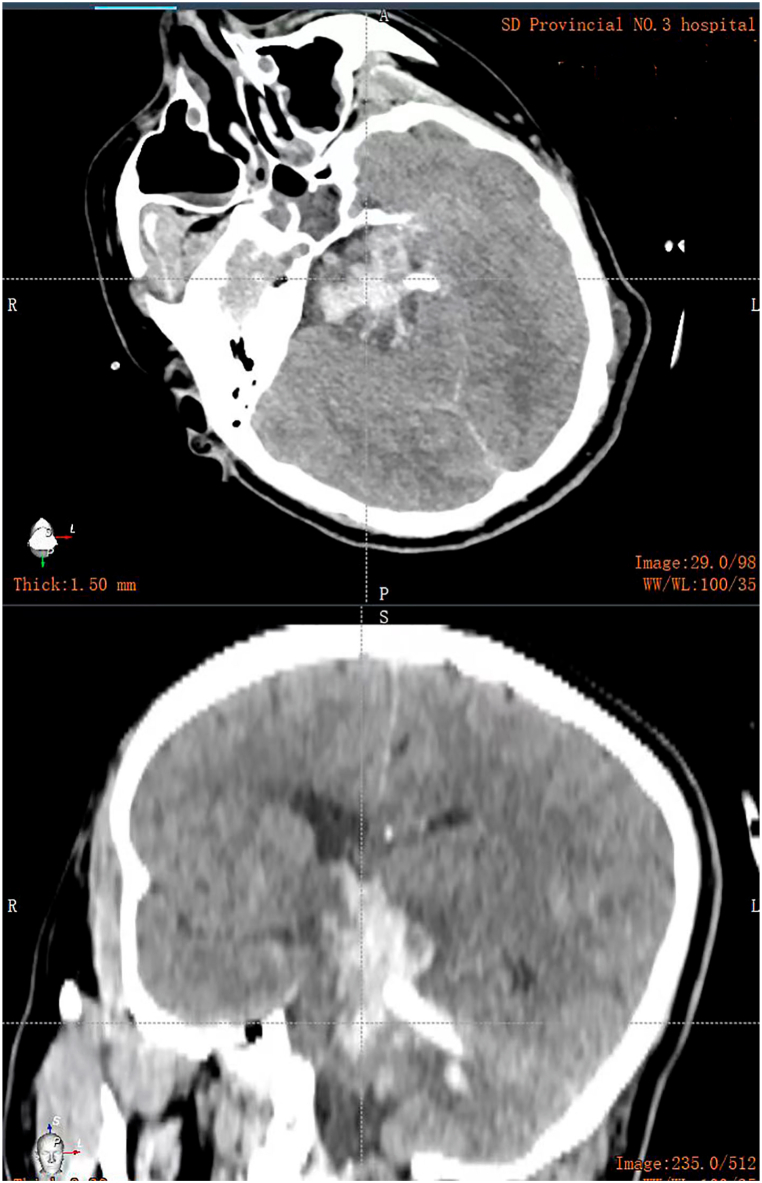
Immediate postoperative CT of case 3.

**Fig. 9:**
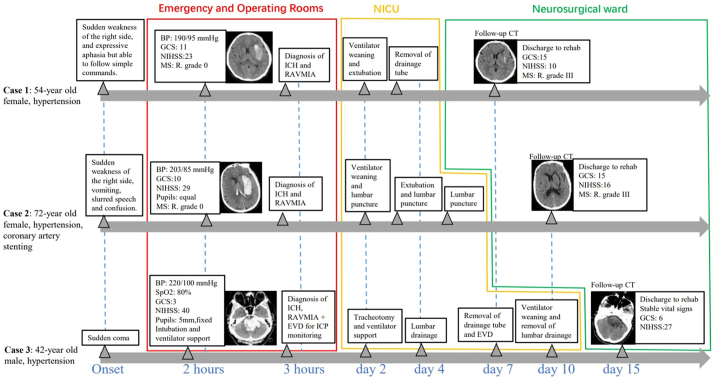
Timelines of the three cases.

A 42-year-old male patient with a history of hypertension presented with a sudden onset of coma lasting for 1.5 hours. Physical examination revealed a blood pressure of 220/100 mmHg, weak spontaneous respiration, and an oxygen saturation level of 85 %. He was in a deep coma with a GCS score of 3. Both pupils were dilated and fixed at a diameter of 5 mm, and the light reflex was absent. Muscle strength was graded as zero in all limbs. Immediate endotracheal intubation and mechanical ventilation were initiated to support respiratory function. Intravenous antihypertensive medication was also administered. A CT scan showed extensive hemorrhage involving the brain stem and third ventricle (Video 5). Despite being informed about the extremely poor prognosis, the family members expressed a strong desire for surgery due to the patient's young age. The RAVMIA was promptly performed with the patient in a prone position. The entry site was set in the suboccipital paramedian area. The surgical trajectory used a trans-medipeduncle intraparenchymal approach to reach the hematoma (Fig. 7). The hematoma was not layered. Initial endoscopy revealed the predominant colloid-like characteristic of the hematoma (Video 6). Multiple aspirations were performed. Final endoscopy showed a solid-like residual hematoma located at the roof of the hematoma, oriented at 12 o'clock (Video 7). Targeted aspiration and placement of a drainage tube were then performed. External ventricular drainage via the transoccipital route was done for ICP monitoring. A CT scan was performed immediately after the surgery (Fig. 8). The patient regained autonomous breathing after a tracheostomy and weaning from ventilator support, maintaining stable vital signs thereafter. The two drains were removed on postoperative day six without any improvement in the GCS score. However, lower limb muscle strength improved to grade I. A CT scan on postoperative day ten revealed near-complete resolution of the hematoma (Video 8). The timelines of the three reported cases are shown in Fig. 9.

### 2.3 Data collection and statistical analysis

The GCS and NIHSS scores were collected from physical examinations. The hematoma volume was determined using robot software. The EOT (End of Treatment) residual ICH volume was calculated from the last CT scan before drainage removal. The data were analyzed using SPSS Statistics version 22 (IBM Corp., Armonk, NY, USA). Measurement data were expressed as mean ± standard deviation (Mean ± SD) and analyzed using a *t*-test for group comparisons. Categorical data were expressed as rates (%) and analyzed using the Chi-square test. Differences were considered statistically significant at P < 0.05.

## 3 Results

The RAVMIA technique was successfully completed without complications in three cases. The data are shown in Table 1. Compared to robotic MISTIE, RAVMIA did not significantly prolong operative time (20.67 ± 4.04 minutes vs 20.87 ± 5.74 minutes, p = 0.946). However, it significantly increased the intraoperative hematoma evacuation rate (86.6 % ± 1.3 % vs 80.8 % ± 4.1 %, p = 0.003), decreased the EOT residual ICH volume (1.3 ± 1.05 ml vs 5.33 ± 2.95 ml, p = 0.04), and shortened the hospital stay (10.67 ± 4.04 days vs 12.87 ± 4.55 days, p = 0.029) (Table 2).

**Table 1:** The results of RAVMIA.

	Case 1	Case 2	Case 3
Gender/age(y)	Female/54	Female/73	Male/42
Preoperative GCS	11	10	3
Preoperative NIHSS	23	29	40
Hematoma location/shape	Left putamen/long oval	Left basal ganglia/long oval	Brain stem/quasi-circular
Preoperative hematoma volume	30.02 ml	50.27 ml	28.4 ml
Robot registration accuracy	0.9 mm	1.1 mm	0.4 mm
Entry site/approach	Forehead/frontal	Forehead/frontal	Suboccipital paramedian/tans-medipeduncle
Surgical duration	25 minutes	17 minutes	20 minutes
Brain damage	5 mm	5 mm	5 mm
Postoperative hematoma volume	3.8 ml	6.27 ml	4.25 ml
Percentage of evacuation	87.3 %	87.5 %	85 %
Length of ICU stay	2 d	4 d	9 d
Time of bearing drainage tube	2 d	3 d	6 d
NIHSS at discharge	10	16	27

Brain damage: the outer diameter of the trocar.

**Table 2:** Comparison of RAVIA and robotic MISTIE.

	RAVMIA (n = 3)	Robotic MISTIE (n = 15)	t/χ [2]	P
Age (years)	56.33(15.63)	64 (10.49)	−0.814	0.489
Sex (Male)	1 (33.3 %)	8 (53.3 %)	0.4	0.527
Comorbidities				
Stroke	1 (33.3 %)	7 (46.7 %)	0.18	0.671
Hypertension	3 (100 %)	12 (80 %)	0.72	0.396
Coronary heart disease	1 (33.3 %)	3 (20 %)	0.257	0.612
Diabetes	1 (33.3 %)	4 (26.7 %)	0.055	0.814
Antiplatelets	1 (33.3 %)	8 (53.3 %)	0.4	0.527
Preoperative GCS	8 (4.36)	8.93(3.43)	−0.349	0.754
Preoperative NIHSS	30.67(8.62)	27.33(6.21)	0.639	0.578
Hematoma volume (ml)	36.23 (12.19)	32.95 (15.84)	0.403	0.71
Hematoma location			0.055	0.812
Basal ganglia	2 (66.7 %)	11 (73.3 %)		
Brain stem	1 (33.3 %)	4 (26.7 %)		
Time to surgery (h)	2.5(0.5)	7.1 (4.44)	−3.891	0.001
Time of surgery (min)	20.67 (4.04)	20.87 (5.74)	−0.072	0.946
Intraoperative hematoma evacuation rate	86.6 % (1.3 %)	80.8 % (4.1 %)	3.57	0.003
Duration with drainage tube (d)	3.33 (1.53)	4.87 (1.21)	−2.57	0.06
EOT residual ICH volume (ml)	1.3 (1.05)	5.33 (2.95)	−3.39	0.04
Rebleeding	0	1 (6.7 %)	0.212	0.645
Intracranial infection	0	1 (6.7 %)	0.212	0.645
Length of ICU stay (d)	5 (3.16)	3.6 (2.37)	−0.832	0.416
Length of hospital stay (d)	10.67 (4.04)	12.87 (4.55)	−2.35	0.029
NIHSS at discharge	17.67 (8.14)	16.87 (5.68)	0.22	0.83
Mortality at 3 months	0	1 (6.7 %)	0.212	0.645
NIHSS at 3 months	13.33 (8.39)	12.13 (5.01)	0.28	0.78

The values in parentheses represent the standard deviation or percentage.

## 4 Discussion

### 4.1 Dilemma of evidence-based medicine in ICH surgery

Evidence-based medical research on the surgical treatment of ICH faces significant challenges. The STICH clinical trials from 2005 to 2013 did not confirm that conventional craniotomy is superior to conservative treatment [2,3]. Key reasons for these negative results include the strong heterogeneity in ICH among individuals, variation between medical centers due to poor quality control of craniotomy, and surgery-related brain damage offsetting the benefits of rapid hematoma evacuation.

Since then, minimally invasive catheterization techniques (tube or needle) causing only a few millimeters of brain damage have been rapidly and widely adopted. Initial clinical trials have shown that this technique is safe and effective for hematoma evacuation [10,11]. However, because there is no direct visualization of the hematoma, the evacuation efficiency is lower than that of open surgeries. This is an inherent limitation of the technique. To address this, our team has identified factors influencing evacuation efficiency and developed a multivariable predictive function to assess it [12]. We have also identified specific subtypes of hematomas that are more likely to be efficiently drained using this technique [13]. Despite these efforts, the MISTIE III clinical trial in 2019 showed that this technique did not improve one-year prognoses for ICH patients [4]. The MISTIE technique has robust quality control and minimal variation among medical centers. Factors contributing to the negative outcome include inconsistent accuracy of catheterization trajectory and an inability to achieve evacuation efficiency comparable to open surgeries within narrow channels.

Sheath-mediated endoscopic surgery (ES) has since gained recognition for its efficient evacuation of hematoma under direct visualization, though it causes centimeter-level brain damage. Our team has developed novel ES approaches for medial thalamic hemorrhage involving the third ventricle and severe intraventricular hemorrhage [14,15]. The recently published ENRICH clinical trial validated the efficacy of ES in improving patient prognosis for lobar hemorrhages. However, the efficacy for basal ganglia hematomas remains unconfirmed [16]. This may be due to greater brain tissue damage caused by using a centimeter-diameter sheath for deep hematomas compared to superficial ones.

### 4.2 Emerging minimally invasive techniques

The use of neurosurgery robots has improved the precision of minimally invasive catheterization [17]. However, they have not changed the non-visualized nature of the MISTIE technique. The low evacuation efficiency remains a significant drawback. The goal of surgical treatment for ICH should be to achieve rapid, maximal hematoma evacuation with minimal brain damage while ensuring safety.

The RAVMIA technique has achieved visualized surgery with millimeter-level brain damage. The safety of its core technique, minimally invasive catheterization, has been proven in MISTIE clinical trials [4,10,11]. RAVMIA can be seen as a modified MISTIE technique, with two main features: 1. Use of Robots: Instead of stereotaxis, robots are used. Both methods allow accurate target control, but the robot's trajectory control is superior [18]. The robot ensures the surgical trajectory aligns with the long axis of the hematoma. 2. Contact-Visible Endoscopy: This is used for “intraoperative monitoring” to show the characteristics, depth, and orientation of the residual hematomas in real-time. This helps determine how deep and where to aspirate the solid-like residual hematoma during surgery, improving evacuation efficiency. The learning curve for the RAVMIA technique is short. With proper training, it can be mastered quickly, making it easy to promote clinically.

Another millimeter-level visualized approach is the stereotactic intracerebral hemorrhage underwater blood aspiration (SCUBA) technique. This method uses water environment endoscopy with the Apollo or NICO Myriad aspiration device for visualized hematoma evacuation [19,20]. Its intraoperative hematoma evacuation rate can reach 88.1 % [21], similar to RAVMIA. It allows surgeons to “see” and “do” simultaneously. However, the clarity in a water environment is not as good as traditional ES or contact-visible endoscopy. Unlike the immobilized 5mm trocar used in RAVMIA, the 6.3mm trocar in SCUBA requires slight movement within brain tissue, needing a centimeter-level skin incision and bone hole [22]. SCUBA also requires sufficient operating space in the water environment, making it unsuitable for small hematomas like brain stem hemorrhage.

### 4.3 Limitation of the study

Compared to traditional sheath-mediated ES, RAVMIA reduces brain damage from centimeters to millimeters. However, it cannot “see” and “do” simultaneously within a narrow 5mm space, unlike traditional ES. This limitation makes effective cauterization hemostasis difficult. The contact-visible endoscope kit, equipped with a corresponding sheath, can transition to traditional ES if active bleeding requires cauterization hemostasis [23]. This was not needed in our reported cases. Table 3 shows a technical comparison of RAVMIA with other minimally invasive techniques.

**Table 3:** Technical comparison of recent minimally invasive procedures.

	Sheath-mediated Endoscopic surgery	Robotic MISTIE	SCUBA	RAVMIA
Hematoma visibility	Yes	No	Yes	Yes
Intraoperative hematoma evacuation rate	80%–100 %	∼70 %	88.1 % ± 12.1 % [21]	86.6 % ± 1.3 %
Intraoperative cautery	Yes	No	Hard	No
Brain damage	10–30mm	4.8mm	6.3mm	5mm
Safety confirmed by RCT	ENRICH [16]	MISTIE [4]	–	MISTIE [4]
Suitable for brain stem hematoma	Yes (skull base approach)	Yes	No	Yes

Brain damage: the outer diameter of the sheath, trocar, or drainage tube.

This study has several limitations. First, the findings are based on a small sample size, and no long-term prognosis study was conducted. Further studies with a larger patient cohort and extended follow-up periods are necessary. Second, the retrospective nature of the case report may introduce recall bias and affect data accuracy. Finally, the effectiveness of this technique has only been studied in patients with long, oval-shaped basal ganglia hematomas and brain stem hematomas. Other types of hematomas, such as anticoagulant-associated ICH, multilobulated, or irregularly shaped ICH, require further research.

## 5 Conclusion

The preliminary application of the RAVMIA technique shows it is safe and feasible for treating long, oval-shaped ICHs in the basal ganglia and brain stem hematomas. It achieves high evacuation efficiency while minimizing invasiveness. Further technical optimization and clinical trials are needed to explore its full potential.

## CRediT authorship contribution statement

**Zhenyu Luo:** Writing – original draft, Data curation. **Chen Li:** Methodology, Formal analysis, Data curation. **Xiaoguang Du:** Writing – original draft, Formal analysis, Data curation. **Tingzhong Wang:** Writing – review & editing, Project administration, Conceptualization.

## Data availability statement

The data that support the findings of this study are available on request from the corresponding author.

## Ethics statement

This study was approved by the ethics committee of our institute. All patient data has been anonymized. Informed consent was obtained from all participants included in this study.

## Funding statement

None.

## Declaration of competing interest

The authors declare that they have no known competing financial interests or personal relationships that could have appeared to influence the work reported in this paper.
